# The AGING Initiative experience: a call for sustained support for team science networks

**DOI:** 10.1186/s12961-018-0324-y

**Published:** 2018-05-18

**Authors:** Tullika Garg, Kathryn Anzuoni, Valentina Landyn, Alexandra Hajduk, Stephen Waring, Leah R. Hanson, Heather E. Whitson

**Affiliations:** 1 Department of Urology, Geisinger Health System, Danville, PA United States of America; 2 Department of Epidemiology & Health Services Research, Geisinger Health System, Danville, PA United States of America; 3 Meyers Primary Care Institute, A joint endeavor of University of Massachusetts Medical School, Reliant Medical Group, and Fallon Health, Worcester, MA United States of America; 4 Section of Geriatrics, Department of Internal Medicine, Yale School of Medicine, New Haven, CT United States of America; 5 Division of Research, Essentia Institute of Rural Health, Duluth, MN United States of America; 6 HealthPartners Institute, Minneapolis, MN United States of America; 7 Duke Aging Center, Duke University, Durham, NC United States of America; 8 Geriatric Research, Education and Clinical Center, Durham Veterans Affairs Medical Center, Durham, NC United States of America; 9 Medicine (Geriatrics) & Ophthalmology, Duke University Center for the Study of Aging, DUMC box 3003, Durham, NC 27710 United States of America

**Keywords:** Geriatrics, Multiple chronic conditions, Team science, Science of team science

## Abstract

Team science, defined as collaborative research efforts that leverage the expertise of diverse disciplines, is recognised as a critical means to address complex healthcare challenges, but the practical implementation of team science can be difficult. Our objective is to describe the barriers, solutions and lessons learned from our team science experience as applied to the complex and growing challenge of multiple chronic conditions (MCC). MCC is the presence of two or more chronic conditions that have a collective adverse effect on health status, function or quality of life, and that require complex healthcare management, decision-making or coordination. Due to the increasing impact on the United States society, MCC research has been identified as a high priority research area by multiple federal agencies. In response to this need, two national research entities, the Healthcare Systems Research Network (HCSRN) and the Claude D. Pepper Older Americans Independence Centers (OAIC), formed the Advancing Geriatrics Infrastructure and Network Growth (AGING) Initiative to build nationwide capacity for MCC team science. This article describes the structure, lessons learned and initial outcomes of the AGING Initiative. We call for funding mechanisms to sustain infrastructures that have demonstrated success in fostering team science and innovation in translating findings to policy change necessary to solve complex problems in healthcare.

## Background

The National Institutes of Health defines team science as “*a collaborative effort to address a scientific challenge that leverages the strengths and expertise of professionals trained in different fields*” [1]. Team science is emerging as an important method to bring together diverse skillsets and data to solve complex clinical problems. Conceptually, team science holds great promise to accelerate translation of research findings into creative clinical solutions; however, the practical implementation of collaborative research brings many challenges, ranging from geographic dispersion to time-intensive infrastructure building [2]. The objective of this paper is to describe the barriers, solutions and lessons learned from our team science experience as applied to the complex and growing challenge of multiple chronic conditions (MCC). The Advancing Geriatrics Infrastructure and Network Growth (AGING) Initiative was funded by the National Institute on Aging in 2014 for a period of 3 years to develop team science infrastructure to propel MCC research.

We will describe the challenges and opportunities of bringing together two culturally different research/healthcare networks as well as specific barriers and solutions that the AGING Initiative has encountered. We share these experiences for the purpose of providing a framework for use by investigators, institutions or agencies keen to foster team science around other complex problems. Finally, we discuss strategic support of junior investigators, which is necessary to develop the next generation of team scientists. We also discuss the need for mechanisms that not only create infrastructure to stimulate new team science, but can sustain and perpetuate successful networks, as well as translate research findings into policy.

## Multiple chronic conditions overview

MCC has been referred to as the ultimate geriatric syndrome [3]. The National Quality Forum defines MCC as the concurrent presence of “*two or more chronic conditions that collectively have an adverse effect on health status, function, or quality of life and that require complex healthcare management, decision-making, or coordination*” [4]. People with MCC now make up over one-quarter of the United States population, and more than half of older Americans live with three or more chronic conditions [5, 6]. Due to advances in medical care and public health that have allowed people to live longer with chronic disease, the number and proportion of patients with MCC is on the rise [7].

The rising prevalence of MCC is concerning because people with MCC are more likely to see multiple providers and receive five or more medication prescriptions [8, 9]. MCC is a risk factor for fragmented or incomplete care [10, 11]. Individuals with MCC suffer high rates of complications and adverse events [4, 12]. As a result, MCC is associated with staggering healthcare costs – two-thirds of Medicare beneficiaries with MCC account for approximately 96% of expenditures [13–15]. Over the past decade, MCC has become a priority area for health-focused government agencies, including the Centers for Disease Control and Prevention, Office of the Assistant Secretary of Health, Agency for Healthcare Research and Quality, and the National Institute on Aging (NIA). Because patients with MCC are heterogeneous and the field, by definition, cuts across many medical specialties and health services, the study of MCC is ripe for the application of team science.

## Bringing together two large research/healthcare networks

The purpose of the AGING Initiative is to create a national resource to develop and advance team science-based research and policy focused on older adults with MCC. The AGING Initiative brings together expertise and leadership from two major research and healthcare networks – the Health Care Systems Research Network (HCSRN) and the Claude D. Pepper Older Americans Independence Centers (OAIC).

Since 1994, the HCSRN, formerly known as the HMO Research Network, has conducted research to improve healthcare delivery and population health by applying the learning health system concept. The HCSRN is made up of 20 non-profit healthcare delivery systems with embedded research departments whose scientists are dedicated to public domain research. Members are typically large, integrated healthcare delivery systems with defined patient populations, and access to electronic health records and administrative data. More than 1900 faculty and staff work in HCSRN research centres, and the combined patient population exceeds 28 million.

The HCSRN specialises in multi-site studies using electronic health record data organised in a data model standardised across sites. The Virtual Data Warehouse facilitates multi-site research while protecting patient privacy and proprietary health practice information. The Virtual Data Warehouse is virtual in that each HCSRN member organisation maintains control of its own electronic health record data via a ‘distributed’ or ‘federated’ model, so it is not a central database. Administrative, clinical and claims data are mapped to a common set of data standards at each site, and a library of programmes support the extract/transfer/load process as needed for a given research study.

In contrast, the OAIC Program is a NIA-funded consortium of centres of excellence in geriatrics research and education that focus on maintaining and restoring functional independence in older adults. There are currently 15 active OAICs located at academic medical centres distributed across 11 states in the United States. Each OAIC is governed internally, with oversight by External Advisory Committees and the NIA, and they often collaborate on multi-site studies such as Lifestyle Interventions and Independence for Elders and Strategies to Reduce Injuries and Develop Confidence in Elders [16, 17]. HCSRN sites have also participated in multi-site projects led by the OAIC.

**Fig. 1:**
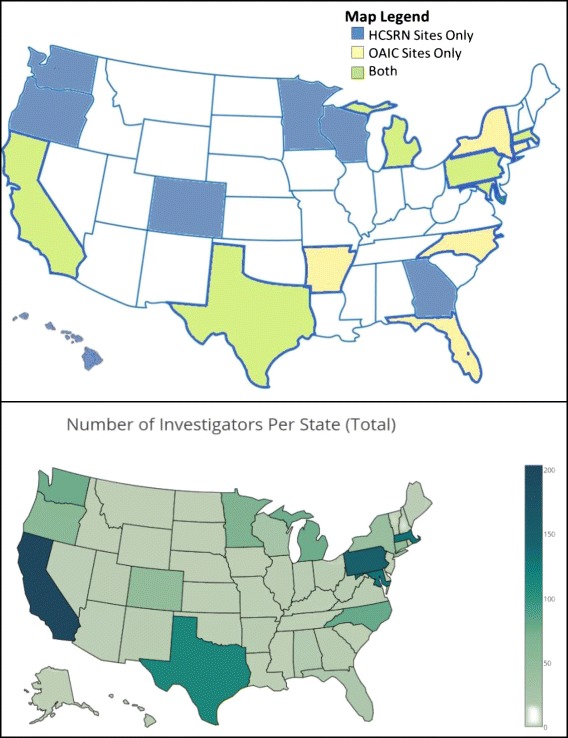
**a** Geographic dispersion of Healthcare Systems Research Network (HCSRN) and Claude D. Pepper Older Americans Independence Centers (OAIC) sites. **b** Density of investigators

In addition to providing outstanding educational and operational support for aging-related research, the OAICs are an important repository for datasets and biospecimens. Housed at Wake Forest School of Medicine, the Integrated Aging Studies Databank and Repository holds extensive data, particularly measures of physical function, body composition and quality of life as well as biological samples (serum, plasma, DNA, skeletal muscle) and imaging, from over 3100 older participants enrolled in over 30 different studies, many with data/samples from before and after an intervention. The Lifestyle Interventions and Independence for Elders study, a multi-site trial of lifestyle interventions aimed at preserving mobility in older adults, offers access to coded datasets, as well as data dictionaries and individual study protocols [16–18]. Figure 1a depicts the geographic distribution of HCSRN and OAIC sites. Given the wide physical distances between sites and investigators, a team science infrastructure was necessary to forge new, cross-cutting research partnerships and teams to effectively address the problem of MCC.

The unique characteristics of these two large networks provides an opportunity to bridge the research-to-policy gap. By teaming with the HCSRN, investigators from both networks can translate findings directly by implementing effective tools and interventions into large, real-world practice networks that impact millions of lives.

## Creating an organisational structure to foster team science

**Fig. 2:**
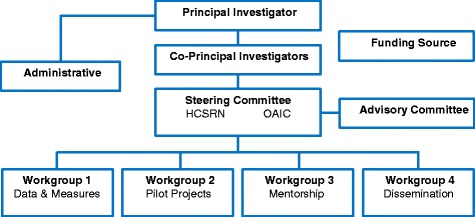
AGING Initiative organisational chart

In order to bridge HCSRN and OAIC sites and investigators, the AGING Initiative founders designed an organisational structure to promote synergy and foster team science (Fig. 2). Four workgroups were established, namely Data and Measures, Pilot Projects, Mentorship, and Dissemination. The workgroups are overseen by a Steering Committee and an external Advisory Committee. Workgroups report monthly to the Steering Committee, which provides guidance and feedback on workgroup activities. The Steering Committee and external Advisory Committee hold a yearly ‘reality check’ meeting to review quantitative and qualitative outcomes, assess whether the aims of the Initiative are being met, and guide future efforts to advance MCC research. Two research coordinators (KA and VL) are critical to the day-to-day operations of the Initiative.

The leadership of each workgroup was intentionally designed to be dyadic with one co-lead from the HCSRN and one from the OAIC in order to enhance communication between the two networks. Each workgroup is also governed by a set of specific aims. The aim of the Data and Measures Workgroup is to collaboratively develop a framework and procedures to enhance the existing HCSRN and OAIC data infrastructure to advance interdisciplinary research on older adults with MCC. This workgroup was felt to be important as one of the idiosyncrasies of MCC research is a lack of standardised definitions and techniques for measurement. The aim of the Pilot Projects Workgroup is to support interdisciplinary pilot projects that optimise HCSRN and OAIC resources and foster collaboration between HCSRN and OAIC investigators to address questions relevant to data validity, comparative effectiveness, health outcomes, health disparities and costs of care in older persons with MCC. The aim of the Mentorship Workgroup is to identify and mentor junior faculty with a research focus on older adults with MCC. Finally, the aim of the Dissemination Workgroup is to disseminate multidisciplinary research approaches and findings relating to the science of MCC to the larger scientific community.

## Perpetuating community through dissemination

The dissemination of programmes, scientific findings and successes resulting from a team science network is important to growing and coalescing a community of investigators around a complex research problem. This is the purpose of the Dissemination Workgroup in the AGING Initiative. Building and expanding a community of MCC researchers extends the reach of the science conducted through the Initiative. Examples of dissemination activities include a quarterly newsletter, a webinar series and presence at national meetings.

To date, 10 newsletters have been distributed (4 in 2015, 4 in 2016, 2 in 2017), with their content including (1) upcoming events such as conferences, workshops and webinars, (2) resources and funding opportunities, (3) recent publications, often with a HCSRN or OAIC investigator as a principal investigator (PI) or co-PI, and (4) featured investigators. The webinar series raises the visibility of research teams within the MCC community and the broader HCSRN and OAIC networks. The workgroup also manages an investigator database and the front-facing network website.

The AGING Initiative has been highly visible at national scientific meetings, including the annual meetings of each network and scientific meetings relating to geriatrics. Since September 2014, the AGING Initiative has contributed 45 presentations at national scientific meetings, including poster presentations and symposia. In addition to these presentations, the AGING Initiative has organised five ancillary meetings and/or workshops at the HCSRN and OAIC annual meetings with increasing attendance each year.

## Barriers and solutions to success of the team science initiative

Many research teams experience a four step process during team development that involves forming, storming, norming and performing [19]. Merging the different cultures of two large research networks incorporated aspects of each of these steps, especially storming and norming. Storming involves establishing roles and responsibilities, communication and processes. In the norming step, team members start to gain comfort and trust in each other and begin to work together efficiently.

In this section, we present barriers faced during implementation of the AGING Initiative and solutions that were developed to overcome each barrier. A summary of the barriers and solutions is presented in Table 1.

**Table 1:** Facilitators and barriers to success of the team science initiative

Barriers	Solutions
Cultural differences between the original research networks	Opportunities for in-person networking
Expanding reach to include diverse perspectives and expertise	Innovative ways to facilitate ‘matchmaking’
Limited salary support to maintain network infrastructure	Enhance efficiency by overlapping workgroup activities and funding a coordinator position
Reliance on soft money limits ability to undertake small projects that build research teams	Funding competition to catalyse new project-based collaborations
Time and energy displaced toward sustaining funds for team science network	Not yet solved

### Barrier: cultural differences between the original research networks

Each of the two networks – HCSRN and OAIC – has their own traditions, processes and workplace cultures. There were perceptions of cultural differences and lack of knowledge regarding each other’s organisational structures and functions. Initially, some cultural differences required education and discussion to resolve. The AGING Initiative Steering Committee devoted much of its first meeting to presentations and discussions intended to help members fully understand the structural arrangement, funding flow, types of data available within each network, and methods to integrate diverse datasets for research. For example, since individual HCSRN sites have control over their own electronic health record data, establishing data sharing agreements and secure file sharing portals has been a critical part of each initial collaborative interaction. The two networks also found many similarities, including the ever-present challenges of the current funding environment and interest in supporting junior investigators. The HCSRN and OAIC, while culturally different, bring complementary expertise, resources, and existing structures that the AGING Initiative now brings to bear on gaps related to MCC research.

### Solution: opportunities for in-person networking

In-person meetings are critical to increasing cohesiveness between members of culturally and geographically disparate team science initiatives [20]. The AGING Initiative provided multiple opportunities for face-to-face interactions. The Steering Committee holds an annual all day meeting at the NIA, as well as shorter, less formal networking events at the annual HCSRN and OAIC annual meetings. Annual meetings of each network provided additional opportunities for members affiliated with each network to become more familiar with individuals and systems in the other network. The Initiative funds one or two investigators from OAIC to attend the HCSRN Annual Meeting and vice-versa to engage investigators from each other’s networks, forge new connections and generate interest from new investigators. Collaborative teams formed through Pilot Project research grants sponsored by the AGING Initiative resulted in strong investigator interactions and strengthened group culture. With in-person meetings and education for each network, investigators from both the HCSRN and OAIC’s found more similarities than differences between the two workplace cultures.

### Barrier: expanding reach to include diverse perspectives and expertise

Successful team science results from intentional efforts to bring together individuals with outside expertise and diverse perspectives to solve complex problems in new ways. MCC research generally draws from experts with geriatrics-focused clinical and research expertise; however, the study of MCC has broad applicability to many medical and surgical subspecialties that treat older adults. Additionally, the study of MCC frequently requires interdisciplinary creation of new research methodology, approaches or instruments due to the complexity of the problem.

At implementation, the Initiative faced challenges in expanding the network to include individuals outside of established MCC researchers and clinicians. For example, many of the first wave Pilot Project applicants and awardees were mentees of AGING Initiative Steering Committee members, and would have likely formed research collaborations without the assistance of a team science network. Identifying and including specialists and subspecialists outside of geriatrics and internal medicine was also a barrier. After identifying this barrier following the first round of funding, the Initiative increased efforts to recruit broadly by circulating newsletters, hosting open interest group meetings and symposia at national meetings, sending out fliers, and widely advertising the webinar series.

### Solution: innovative ways to facilitate ‘matchmaking’

An important component of team science is that investigators go outside their usual circles to identify resources and skillsets needed to ‘round out’ the team’s expertise. Building functional teams capable of pioneering new directions in science requires a certain amount of ‘matchmaking’. The AGING Initiative has been successful in building a diverse nationwide network of over 1600 investigators with relevant expertise, who are now more aware of MCC and increasingly engaged in MCC research. Figure 1b demonstrates the geographic areas of the United States where investigators are based. California, Pennsylvania and Massachusetts had the highest density of investigators. The majority of investigators in the AGING Initiative Network were from HCSRN or OAIC sites, with smaller percentages coming from the National Institutes of Health, Department of Veterans Affairs, and the Agency of Healthcare Research and Quality. Many of these individuals learned about the AGING Initiative through dissemination efforts or word of mouth and requested to be included through the listserve.

A new effort to expand on investigator matchmaking is the addition of a ‘classifieds’ section to the quarterly AGING Initiative newsletter. Investigators seeking individuals with specific methodologic expertise, datasets or collaborators for upcoming grant submissions may submit a classified advertisement to solicit assistance from the investigator community.

### Barrier: limited salary support to maintain network infrastructure

In a large, geographically disparate and culturally diverse team science network such as the AGING Initiative, finding budget-friendly ways to maintain network infrastructure (e.g. day-to-day operations, electronic resources, network-wide communication) is important to the initial success and long-term health of the network. Budgets for team science networks may be limited or dispersed across many investigators and organisations, and infrastructure maintenance budgets may be limited from the outset at the proposal phase.

### Solution: enhance efficiency by overlapping workgroup activities and funding a coordinator position

During the AGING Initiative implementation process, there has been constructive overlap and cross-talk across workgroups. For example, the Mentorship Workgroup refers junior investigators to the Pilot Project Workgroup and vice versa to make connections with senior investigators or across the networks. The Dissemination Workgroup’s webinar series features completed Pilot Projects and provides an educational overview of a broader MCC-related topic by a recognised expert. These types of ‘double-duty’ teamwork activities maximise limited investigator time and financial resources.

To facilitate efficient execution of overlapping activities across the organisational structure, the Initiative made a conscious decision to budget for a full-time coordinator position. The coordinator partners with the Initiative PI’s and workgroup co-leads to organise connections and meetings, collect outcomes data, manage electronic resources (e.g. webpage, newsletter, listserve), and perform other tasks vital to organisational structure and success.

### Barrier: reliance on soft money limits ability to undertake small projects that build research teams

Building new and effective interdisciplinary research teams may require demonstration of feasibility to team members and funders. Successful completion of simple, small projects allows research teams to develop relationships and scientific cohesion in a low-risk environment. Propelled by initial success from smaller projects, the teams may evolve towards larger, full-fledged research programmes. However, research centres within the HCSRN and OAIC networks are highly reliant on soft money from larger grants to maintain investigators and resources, limiting investigator interest and bandwidth to engage with new research teams.

### Solution: funding competition to catalyse new project-based collaborations

The AGING Initiative instituted a Pilot Project programme as the main vehicle for forming research teams and gauging their effectiveness. These $40,000 1-year grants require at least one investigator from the OAIC and one from the HCSRN on the research team, as well as a junior investigator. At completion of the funding period, the goal is to demonstrate that the research team could successfully execute a project, and then apply the preliminary data and findings towards larger grants to advance MCC science.

Some challenges were noted in the first funding cycle, which were subsequently corrected with feedback from Pilot Project awardees and the Steering Committee. For example, in the initial cycle, research teams had to be delineated prior to submission of the application. In subsequent cycles, instead of having the research team in place at the time of application, a letter of intent was instituted and the Pilot Projects Workgroup offered a ‘matchmaking’ service to connect investigators with similar interests.

To date, four cycles of Pilot Project funding have occurred. A total of 54 applications were received and 13 applications received funding (Table 2). Completed projects are presented to the larger community through the previously discussed webinar series, hosted by the Dissemination Workgroup. The webinars serve as a platform for research teams to raise the visibility of their work with a national network of investigators and other relevant stakeholders, which enhances the value of the money invested in each project. The webinars are held at least quarterly and have been well attended.

**Table 2:** AGING pilot project funding cycles

Cycle	Number of applications received	Number of applications funded	Titles of funded projects	Disciplines
1 (2015)	12	3	1. Diabetes, Dementia, & Multiple Chronic Conditions in Males with Hip Fracture^a^ 2. Multimorbidity & Outcomes in Patients with Implantable Cardioverter Defibrillator^a^ 3. Advance Care Planning Practices in Caring for Vulnerable Elders with Multiple Chronic Conditions^a^	Geriatrics Biostatistics Nephrology Internal Medicine
2 (2015)	10	3	1. Does the Medicare Preventive Visit Coverage Benefit Seniors with Multiple Chronic Conditions?^a^ 2. COPD & Average Attributable Fraction from MultiplE Chronic Conditions (CAAFE)^b^ 3. Electronic Medical Record (EMR) Predictors of Undiagnosed Dementia^a^	Geriatrics Biostatistics Pulmonary/Critical Care Psychiatry
3 (2016)	17	4	1. Multiple Chronic Conditions and Mortality in Older Adults with Superficial Bladder Cancer^a^ 2. Advance Care Planning in Older Adults with Multiple Comorbid Conditions Undergoing High-Risk Surgery^b^ 3. Multiple Chronic Conditions, Frailty, and Mobility/Functional Impairment in Older Adults with Heart Failure^a^ 4. Use of the New Comorbidity Index in the Veteran Elderly to Evaluate the Risk of Death or End Stage Renal Disease After Inpatient Acute Kidney Injury^b^	Urologic Oncology Medical Anthropology Geriatrics Biostatistics Internal Medicine Nephrology Epidemiology Cardiovascular Disease
4 (2017)	15	3	1. Identification of Patient Subgroups in Hospital Readmissions Through Visual Analytics^b^ 2. Assessing the Benefits and Harms of Triple Antithrombotic Therapy in Medically Complex Older Adults with Comorbid Myocardial Infarction and Atrial Fibrillation^b^ 3. Development of a CKD Discordance Index to Identify High Healthcare Utilization^a^	Geriatrics Biostatistics Cardiovascular Disease Nephrology Biomedical Informatics

^a^Indicates Principal Investigator from Healthcare Systems Research Network site

^b^Indicates Principal Investigator from Claude D. Pepper Older Americans Independence Centers site

### Barrier: time and energy displaced towards sustaining funds for team science network

The funding received for the AGING Initiative has been critical to the genesis and success of this team science network. However, while funders and funding mechanisms for team science network infrastructure are burgeoning, overall funding remains limited. As we approach the end of the initial funding term, the AGING Initiative leadership has been identifying new ways to sustain and expand existing infrastructure. Innovations in team science funding and network infrastructure are needed to prevent loss of momentum in the science itself due to diversion of investigator time and energy towards sustaining infrastructure. Currently, the Initiative has not identified a way to overcome this barrier; however, we hope to partner with existing funders to develop solutions.

## Developing the next generation of team scientists

Engaging effectively in team science projects is a critical new skill for success in an increasingly challenging funding environment [2]. The AGING Initiative is highly committed to supporting and developing the talents of junior investigators as a pipeline of future MCC investigators and team science leaders. The Mentorship Workgroup leads this priority; primarily within the context of Pilot Project submissions, it matches mentors and junior investigators with complementary interests to advise on proposal development, discuss the grant application process and provide career advice. Junior investigators are also paired with a senior researcher on the Pilot Project investigator team to provide additional mentorship for the duration of a project. Several of these Pilot Project research teams have continued to work together on subsequent projects relevant to MCC in older adults.

The AGING Initiative supports a yearly forum for young investigators at the annual HCSRN scientific meeting, where pilot grant awardees can present study findings in a poster or podium session. The AGING Initiative provides mentoring and hosts a mock study section at the annual OAIC meeting.

## AGING initiative outcomes

In its first 3 years, the AGING Initiative has demonstrated success in advancing team science for MCC (Table 3). Eighteen manuscripts have already been published or are in press, and an additional 11 manuscripts are in development at this time, all work resulting from teams formed through the AGING Initiative. In addition to manuscripts, several new grant applications have been produced by AGING Initiative research teams. From the AGING Pilot Projects, seven grant applications have been submitted as a result of the six pilot projects in the first two cycles of funding. Additionally, three administrative supplements and one diversity supplement have been funded.

**Table 3:** Summary of AGING initiative outcomes

Type of product	Quantity	Related to funded pilot projects	Led by an early-stage investigator
Grants	17	7	4
Published papers	18	2	1
Submitted manuscripts	11	10	2
Funded pilot projects	13	13	8
Presentations at national meetings	45	14	11
Webinars^a^	18	8	N/A

^a^Average 105 attendees (range 31–370), with additional viewings of online recorded content

## Conclusions

Team science has emerged as an important method to bring together diverse skillsets and data to solve complex clinical problems [21–23]. The AGING Initiative experience demonstrates that, in under 3 years, building a team science platform can expand capacity and catalyse collaboration around a complex health problem. As described in this Commentary, building nationwide infrastructure to foster team science requires understanding different workplace cultures, efficiency in data sharing, thoughtfully designing organisational structure, disseminating to expand a diverse community, engaging the next generation of team scientists, and applying a flexible, iterative approach to create solutions to unanticipated barriers during implementation.

While initial experiences such as ours and others demonstrate the feasibility of team science networks, future work will need to focus on growth and sustenance. The new field of the ‘science of team science’ may provide future insight into how to maximise efficiency and productivity of increasingly complex research teams [24]. Teaching junior investigators and trainees how to engage effectively with research teams will be critical for success in the future, and some tools are currently publicly available to assist in this process [1, 2, 25]. Recognition of team science efforts continues to be a challenge. In most centres, decisions regarding promotion and tenure are generally focused on a single individual’s efforts. These types of models may detract from a researcher’s interest in participating in team science, to the detriment of the field [2]. Some professional organisations such as the American Association for Cancer Research have implemented specific awards to highlight the importance of team approaches in scientific advances [26].

In addition, there is a need to encourage policy-makers to develop solutions to expand and sustain successful team science networks and infrastructure [27]. Investment in team science networks has the potential for payoff on a societal level as limited research resources and monies can be utilised efficiently to identify effective interventions and implement them on a national scale. Coordinated efforts that operationalise team science are different from more traditional project support. When such endeavours are reliant on soft money, the risk remains that early scientific momentum will be lost if funding to support infrastructure lacks sustainability beyond 3 or 5 years. Because team science often takes place on a national and international stage, it is unclear which funding source is best positioned to support team science platforms. In addition to government agencies, professional societies, foundations and philanthropic organisations may play a role in the future. Given both the urgency and complexity of medical challenges our society faces, as well as the recognition that the path to a solution is often facilitated by a team science approach, policy-makers should consider allocating resources to maintain platforms that have demonstrated an ability to enable team science.

## Abbreviations

AGING: Advancing Geriatrics Infrastructure and Network Growth
HCSRN: Healthcare Systems Research Network
MCC: Multiple Chronic Conditions
NIA: National Institute on Aging
OAIC: Claude D. Pepper Older Americans Independence Centers
OASH: Office of the Assistant Secretary of Health
PI: Principal Investigator
