# Correction to: The AGING Initiative experience: a call for sustained support for team science networks

**DOI:** 10.1186/s12961-019-0429-y

**Published:** 2019-05-20

**Authors:** Tullika Garg, Kathryn Anzuoni, Valentina Landyn, Alexandra Hajduk, Stephen Waring, Leah R. Hanson, Heather E. Whitson

**Affiliations:** 1 Department of Urology, Geisinger Health System, Danville, PA USA; 2 Department of Epidemiology & Health Services Research, Geisinger Health System, Danville, PA USA; 3 Meyers Primary Care Institute, A joint endeavor of University of Massachusetts Medical School, Reliant Medical Group, and Fallon Health, Worcester, MA USA; 4 Section of Geriatrics, Department of Internal Medicine, Yale School of Medicine, New Haven, CT USA; 5 Division of Research, Essentia Institute of Rural Health, Duluth, MN USA; 6 HealthPartners Institute, Minneapolis, MN USA; 7 Duke Aging Center, Duke University, Durham, NC USA; 8 Geriatric Research, Education and Clinical Center, Durham Veterans Affairs Medical Center, Durham, NC USA; 9 Medicine (Geriatrics) & Ophthalmology, Duke University Center for the Study of Aging, DUMC box 3003, Durham, NC 27710 USA

**Correction to: Health Res Policy Syst (2018) 16:41**

**https://doi.org/10.1186/s12961-018-0324-y**

After publication of the original article [1], it came to the authors’ attention that a funding source was omitted. This Correction article shows the updated Funding section.
